# Differential Foraging of Indigenous and Exotic Honeybee (*Apis mellifera* L.) Races on Nectar-Rich Flow in a Subtropical Ecosystem

**DOI:** 10.3390/insects11040254

**Published:** 2020-04-19

**Authors:** Abdulaziz S. Alqarni

**Affiliations:** Department of Plant Protection, College of Food and Agriculture Sciences, King Saud University, Riyadh 11451, P.O. Box 2460, Saudi Arabia; alqarni@ksu.edu.sa

**Keywords:** subtropical apiculture, *Acacia gerrardii*, *Apis mellifera jemenitica*, bee plants, nectar and pollen collection, desert beekeeping

## Abstract

In the subtropics, agricultural activities such as beekeeping are greatly influenced by environmental challenges. In the desert of Central Arabia, honeybees forage on limited prairies that are affected by adverse weather conditions. Bee colonies reduce their field activities during extremely hot-dry-windy weather. This study investigated whether nectar-rich melliferous flora enhance the field activities of two honeybee subspecies, *Apis mellifera jemenitica* (indigenous) and *A. m. carnica* (exotic), despite the presence of severe weather conditions. The foraging and pollen-gathering activities of the two subspecies were evaluated on Acacia trees (*Acacia gerrardii* Benth.), a common subtropical, summery endemic bee plant, in the central desert of the Arabian Peninsula. The native colonies were significantly (*p* < 0.001) more active foragers than the exotic colonies (109 ± 4 and 49 ± 2 workers/colony/3 min, respectively). Similarly, the native colonies recruited significantly (*p* ˂ 0.01) more active pollen-gathering bees than the imported colonies (22 ± 1 and 7 ± 1 workers/colony/3 min, respectively). Furthermore, far more food was collected by the indigenous colonies than by the exotic colonies, and a higher portion of all field trips was allocated to pollen gathering by the indigenous bees than by the imported bees. The nectar-rich Acacia trees reduced the negative effects of hot-dry-windy weather. More research on honeybee colonies operating in the subtropical conditions of Central Arabia is needed, especially regarding heat tolerance mechanisms and effects on queen and drone fertility.

## 1. Introduction

Subtropical regions are usually situated between latitudes of 20 and 40 degrees in both hemispheres, where most of the rising economies are found. Beekeeping is a promising profession for low-income individuals and families. Productive beekeeping may serve as a reliable indicator of economic biodiversity and viable ecosystems [1]. In Central Arabia, honeybee plants are limited and found in scattered valleys and oases. Beekeepers are challenged to find fruitful resources of nectar and pollen for their bee colonies. The foraging and pollen-gathering activities of honeybee colonies are governed by many ambient environmental conditions [2,3,4], including forage [5,6], temperature [7,8,9,10,11], relative humidity [12] and wind speed [13]. Additionally, the honeybee genotype greatly influences foraging activities [14,15]. The rates of foraging and pollen gathering reflect the performance and productivity of honeybee colonies under certain circumstances [3].

Acacia (*Acacia gerrardii* Benth.) trees are melliferous plants restricted to Africa and the Middle East. Their benefits, which include use in human fodder, stock pasture, traditional medicine, and honeybee forage, are highly valuable within their ecosystems [16,17]. These trees represent a major honeybee forage source [18] and support one of the most consumer-preferred honey sources in Saudi Arabia [19]. Their maximum flowering period occurs between mid-June and the end of July, during the hottest time of the year in Central Arabia. Despite this environmental challenge, one tree may produce 36 kg of nectar (evaluated as total soluble solids) per season [20]. In addition to honeybees, many flower visitors visit the *A. gerrardii* tree flower heads. Megachilid bees ranked first before honeybees in the zoophily of Acacia flower heads [21], and considerable yield of Acacia honey is produced by honeybee colonies, despite the hyper hot-dry weather conditions that accompany Acacia flowering [22].

Although honeybees can forage for nectar and pollen in a wide range of climates, they evidently reduce their field activities during severe weather conditions [9,23]. However, different bee subspecies respond differently to such circumstances [14].

Beekeeping in Saudi Arabia is practiced on two honey bee subspecies, the native *A. m. jemenitica* and the exotic *A. m. carnica*. The need to import *A.m. carnica* is due to the fact that the native bee population is not sufficient to meet the required colonies for beekeeping activity in the country. Although the native bees always showed higher values in bee population, both subspecies produced similar honey yield at the end of the season [22,24]. However, the exotic colonies population declined and diminished, unlike the native colonies, which stay healthy for the next flowering season. Therefore, some beekeepers used to obtain new exotic bee colonies for each flowering season.

Data on honeybee colony activities throughout the *A. gerrardii* flow, which corresponds to remarkably harsh weather conditions, are lacking. Indeed, how this rich bee forage affects the activities of bee colonies under hot, dry, and windy weather conditions needs to be documented.

This study is one element of a major research project investigating how subtropical weather conditions reflect on different key points related to bee colony performance, e.g., nectar secretion dynamics, flowering phenology, pollination ecology, the honey potential of major bee plants [20,21,25,26], the antimicrobial potential of honeys [17], associative learning and heat shock protein expression [27,28], insecticidal-induced changes in the learning of honeybees [29], and the use of geographic information systems (GIS) and remote sensing technologies to determine the optimal forage capacity [30]. The present experiment was conducted to evaluate the rates of outgoing and pollen-gathering foragers during a period of rich nectar flow. Explicitly, to what degree can honeybee colonies enhance their field activities during the flowering of such nectar-rich melliferous flora, despite severe weather conditions? In this context, two honeybee subspecies, *Apis mellifera jemenitica* (indigenous) and the Carniolan honeybee *A.m. carnica* (imported) were compared. The specific purpose of the study was to determine the extent to which adapted and non-adapted honeybee colonies benefit from Acacia nectar and pollen, despite the extremely hot-dry weather conditions of subtropical regions. The outcome of this work will provide information to develop new strategies for maximizing the economic return of beekeeping in the country and to successfully address the complaints of beekeepers about crowded foraging sites, low rates of honey production, and low survival of exotic bee colonies throughout the production season.

## 2. Materials and Methods

### 2.1. Experimental Area

The field work was carried out in Rawdhat-Khoraim oasis, a conserved region in a vast desert northeast of Riyadh, the Saudi capital. The oasis is located at approximately 25°32’ north and 47°17’ east and has an altitude of 1817 feet. Among the tree species in Rawdhat-Khoraim oasis, *A. gerrardii* accounts for a major proportion of the population, as the plant cover of this species is rich in the oasis compared with the neighboring desert [31]. Acacia trees in the oasis are dependent on the estuaries from surrounding areas during autumn, winter, and spring. The flowering season of Acacia trees occurs between May and August [20]. The oasis is a popular site for migrant beekeepers to collect highly valued Acacia honey, locally called "Talh honey". Field investigations were performed from May to August of 2017 to 2018. A pilot study was carried out in 2016 to standardize the methods that were subsequently followed in 2017 and 2018.

The presence of co-flowering forage during the Acacia flowering season was determined in 2012 in a field survey. Only two co-flowering plant species, both present at a low density, were observed: *Ziziphus nummularia* and *Calotropis procera.* Therefore, *A. gerrardii* was the prevalent forage, and desert weather was the predominant weather condition.

### 2.2. Tested Honeybees

The field activities of the following two honeybee subspecies were evaluated and compared during the Acacia flow: *A.m. jemenitica*, the indigenous subspecies of Saudi Arabia, and *A.m. carnica*, a subspecies often imported from Egypt. Five equal-strength colonies (seven frames) of each tested subspecies were used in each season of 2017 and 2018. The selected colonies were reared in modern, wooden Langstroth hives and were housed under a tent in Rawdhat-Khoraim oases one month prior to the commencement of Acacia flowering each year.

### 2.3. Weather Data

Three weather factors have been hypothesized to affect the foraging and pollen-gathering activities of honeybee colonies: temperature (Temp), relative humidity (RH) and wind speed (WS). These factors were documented simultaneously with the monitoring of outgoing and pollen-gathering foragers. Weather factors for 2017 and 2018 were extracted from data from the nearby Weather Station of King Khalid International Airport. The monthly and seasonal means for Temp, RH and WS were calculated.

### 2.4. Outgoing Workers and Incoming, Pollen-Loaded Foragers

The total numbers of foraging (outgoing) and pollen-gathering (incoming loaded with pollen pellets) worker bees over three min were counted weekly. Data were recorded at five fixed times in the day in every tested colony throughout the Acacia tree flowering period in 2017 and 2018 using a counter and a stopwatch. The recording times were the Arabia Standard Time (AST) of sunrise (SR), forenoon (FN), noon (N), afternoon (AN) and sunset (SS), approximately 0530, 0830, 1130, 1430 and 1730 h, respectively [32,33,34,35].

### 2.5. Data Processing and Statistical Analysis

Microsoft Excel 2013 was used to prepare the data for statistical analysis, to calculate averages, standard errors, and percentages of the data and to prepare the graphs. The statistical analysis was performed using SPSS 22. The mean values are followed by the standard errors throughout the article. The normality and homogeneity of the data were first tested using the Kolmogorov–Smirnov and Levene tests, respectively. Heterogeneity was eliminated using square-root transformation. The statistical significance of the examined factors was tested using ANOVA. The means were separated using Duncan’s test (0.5). Correlations between the examined factors were tested for significance using the Pearson correlation coefficient factor (0.5).

## 3. Results

### 3.1. Weather Data

**Figure 1:**
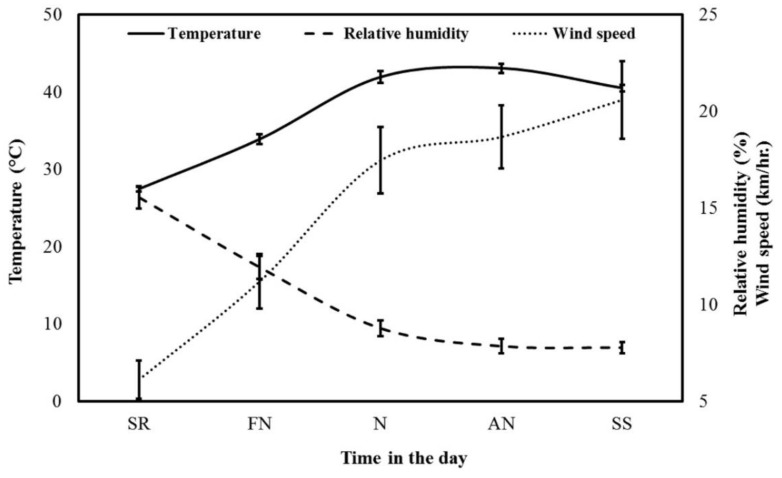
Hourly average values of weather factors during times in the day during the *Acacia gerrardii* flow (May-August) in Central Arabia for the 2017 and 2018 seasons (SR: sunrise; FN: forenoon; N: noon; AN: afternoon; SS: sunset).

**Figure 2:**
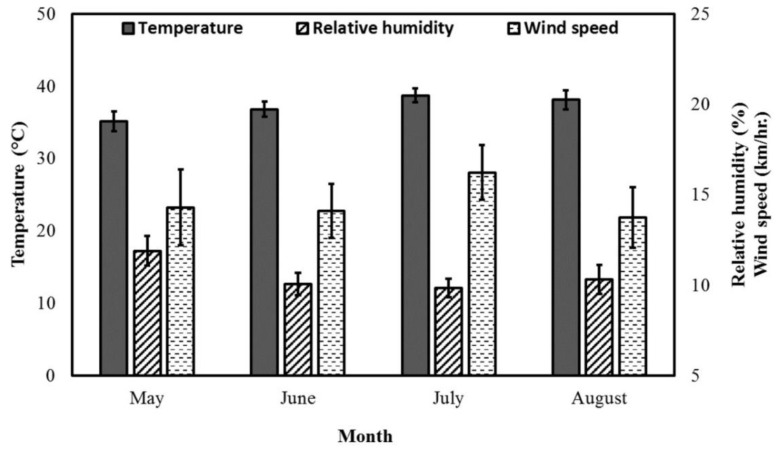
Monthly average values of weather factors during the *Acacia gerrardii* flow (May-August) in Central Arabia for the 2017 and 2018 seasons.

The flowering season of Acacia trees in central Saudi Arabia occurred during extremely hot (mean Temp: 37 °C; range: 23–52 °C), dry (mean RH: 12%; range: 5–21%) and relatively windy (mean WS: 15 km/h; range: 0–41 km/h) diurnal conditions (Figure 1 and Figure 2). Temp values were above 40 °C for more than 45% of the diurnal period during the Acacia flow season. RH values were lower than 10% for more than 50% of the diurnal period during the Acacia flow season. WS was faster than 15 km/h for approximately 38% of the diurnal period during the Acacia flow season. The estimated weather factors showed a clear hourly pattern (Figure 1), but had no obvious monthly pattern (Figure 2).

### 3.2. Foraging Activities

**Figure 3:**
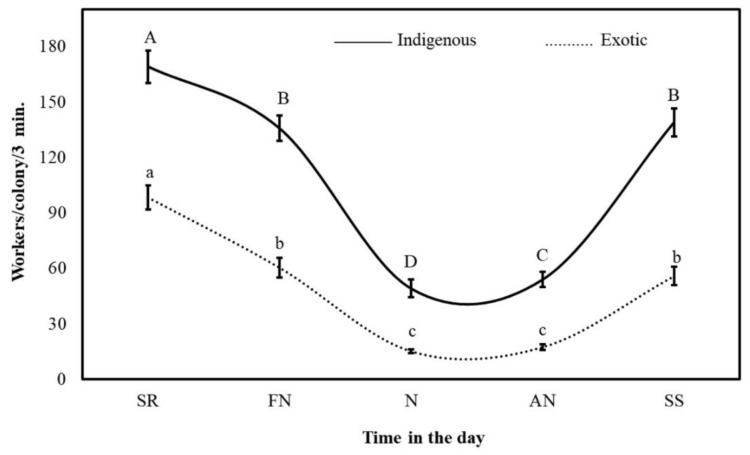
The hourly foraging rates (mean ± SE) of indigenous (*Apis mellifera* *jemenitica*) and exotic (*A.m. carnica*) honeybee colonies during the *Acacia gerrardii* flow (May-August) in Central Arabia for the 2017 and 2018 seasons. (SR: sunrise; FN: forenoon; N: noon; AN: afternoon; SS: sunset). The same letter in the same chart line are not significantly (*p* ˃ 0.05) different.

**Figure 4:**
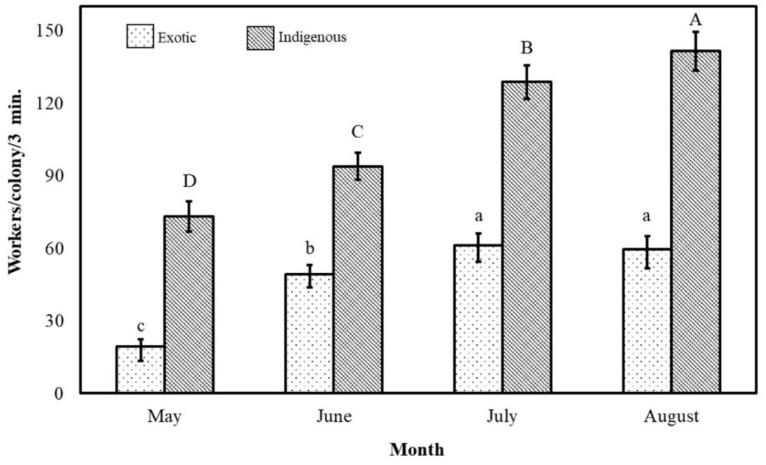
The monthly foraging rates (mean±SE) of indigenous (*Apis mellifera* *jemenitica*) and exotic (*A.m. carnica*) honeybee colonies during the *Acacia gerrardii* flow (May–August) in Central Arabia for the 2017 and 2018 seasons. The same letter are not significantly (*p* ˃ 0.05) different.

The general mean rate of outgoing foragers for all tested bee colonies during the Acacia flow was 79 ± 2 workers/colony/3 min (1578 outgoing bee workers leaving the colony per h). The adapted native bee colonies were significantly (*p* < 0.001) more active than the Carniolan colonies (109 ± 4 and 49 ± 2 workers/colony/3 min, respectively). The number of active foragers in the indigenous colonies was 2.2 times greater than that in the imported colonies, i.e., one native colony recruited more foragers than two Carniolan colonies. During all five specific times in the day, four months, and two seasons tested, the native colonies foraged more actively than the imported colonies. The rate of outgoing foragers varied remarkably according to subspecies, time in the day, month, year, and weather factors (Figure 3 and Figure 4).

The outgoing foraging rates of all colonies in the two subspecies showed clear hourly variations during the Acacia flow. The maximum and minimum foraging rates occurred during the SR and N times, respectively. The foraging rate at SR was 4.2 and 3.8 times greater than that during N and AN, respectively. The colonies performed 33.7%, 24.7%, 8.1%, 9.0% and 24.5% of their daily foraging trips during the SR, FN, N, AN, and SS times, respectively. The peak foraging activity of the native bee colonies occurred at SR (169 ± 9 workers/colony/3 min), while their lowest foraging rate occurred at N (49 ± 5 workers/colony/3 min). Furthermore, the foraging rate of the native colonies at SR was 1.2, 3.4, 3.1 and 1.2 times greater than the foraging rates at FN, N, AN and SS, respectively. These colonies performed 30.9%, 24.8%, 9.0%, 9.9% and 25.4% of their daily foraging flights at SR, FN, N, AN, and SS, respectively (Figure 3).

The maximum outgoing foraging rate for the exotic colonies occurred around SR, while the minimum outgoing foraging rate occurred at N and AN. The foraging flights around SR were 1.6, 6.5, 5.7 and 1.8 times more numerous than the flights during FN, N, AN, and SS, respectively. The imported colonies completed 39.8%, 24.4%, 6.1%, 7.0% and 22.6% of their daily outgoing flights at SR, FN, N, AN, and SS, respectively.

The monthly foraging pattern of all bee colonies of the two subspecies during the Acacia flow was significantly (*p* ˂ 0.01) variable (Figure 4). The maximum foraging rate occurred at approximately the end of the season in August (101 ± 6 workers/colony/3 min), while the minimum rate occurred at the beginning of the season in May (46 ± 4 workers/colony/3 min). The foraging activity during August was 2.2 times greater than that during May (Figure 4).

The native bee colonies foraged at a low rate during the beginning of the Acacia flow (May) and then increased their foraging rates in June and July until reaching their maximum foraging rate in August (142 ± 8 workers/colony/3 min). Their foraging rate during August was 1.9, 1.5 and 1.1 times greater than their foraging rates during May, June and July, respectively. In other words, the monthly foraging rates were approximately 1464, 1878, 2574 and 2830 workers/colony/hr during May, June, July and August, respectively (Figure 4).

The monthly variations in the Carniolan honeybee colonies had a similar course as the above-described variations of the native colonies. However, the rate of outgoing foragers of the imported subspecies was always significantly (*p* ≤ 0.05) less than that of the indigenous subspecies. The foraging rate of the Carniolan honeybee colonies during July was 3.1 greater than that during May, while the foraging rate of these colonies during August was 1.2 times greater than that during June. Their monthly foraging rates were 391, 988, 1226 and 1193 workers/colony/h in July, August, May, and June, respectively (Figure 4).

### 3.3. Pollen-Gathering Activities

**Figure 5:**
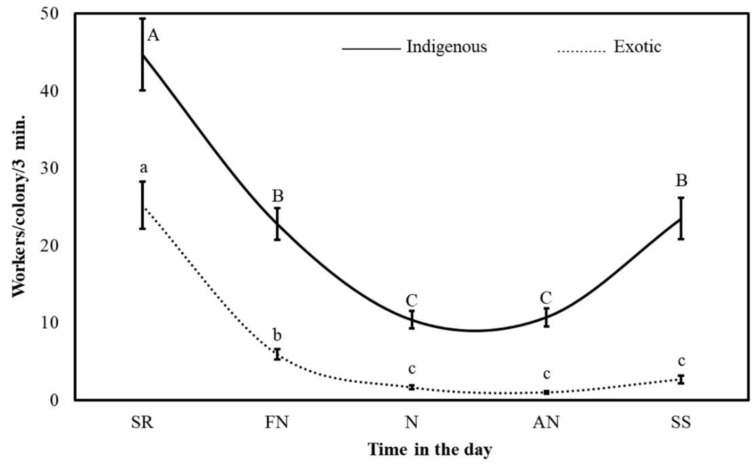
The hourly pollen-gathering rates (mean ± SE) of indigenous (*Apis mellifera* *jemenitica*) and exotic (*A.m. carnica*) honeybee colonies during the *Acacia gerrardii* flow (May–August) in the 2017 and 2018 seasons in Central Arabia. (SR: sunrise; FN: forenoon; N: noon; AN: afternoon; SS: sunset). The same letter in the same chart line are not significantly (*p* ˃ 0.05) different.

**Figure 6:**
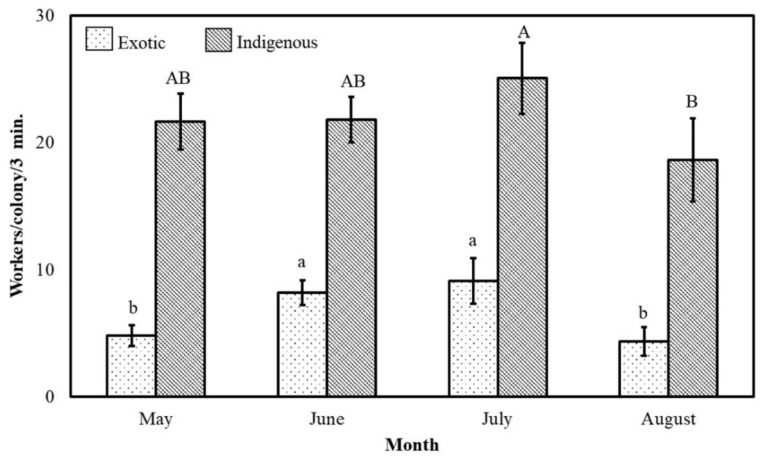
The monthly pollen-gathering rates (mean±SE) of indigenous (*Apis mellifera* *jemenitica*) and exotic (*A.m. carnica*) honeybee colonies during the *Acacia gerrardii* flow (May–August) in Central Arabia for the 2017 and 2018 seasons. The same letter in the same chart line are not significantly (*p* ˃ 0.05) different.

The general mean activity of pollen-gathering foragers during the Acacia flow for both subspecies was 15±1 workers/colony/3 min (range 0–241). Therefore, a honeybee colony received 297 incoming workers with pollen loads (594 pollen pellets) each hour. The native honeybee colonies were significantly (*p* ˂ 0.01) more active pollen gatherers than the Carniolan colonies (22 ± 1 and 7 ± 1 workers/colony/3 min, respectively). Pollen gatherers were 3.1 times more numerous in the indigenous colonies than in the imported colonies, i.e., one native colony gathered more pollen than three Carniolan colonies. The pollen-gathering activity was affected by subspecies, time of day, month, season, and weather factors (Figure 5 and Figure 6).

The native colonies gathered more pollen than the imported colonies during the five times of the day, four months, and two seasons tested. The indigenous colony performed 448 pollen-gathering trips each hour. Simultaneously, the imported colony performed only 145 pollen-gathering trips each hour. At SR, FN, N, AN and SS, 1.8, 3.9, 6.4, 11.1 and 8.8 times more pollen-gathering trips, respectively, were performed by the native colonies than by the Carniolan colonies. The major difference occurred during the AN, when the indigenous colonies had a pollen-gathering rate of 10.7 ± 1.2 workers/colony/3 min, while the imported colonies had a pollen-gathering rate of 1.0 ± 0.2 workers/colony/3 min. The indigenous colonies were 4.5, 2.7, 2.7 and 4.3 times more active pollen gatherers than the imported colonies during May, June, July, and August, respectively (Figure 5 and Figure 6).

The five tested times of day showed three significant (*p* ≤ 0.05) discrete pollen-gathering peaks. The maximum and minimum pollen-foraging rates occurred at SR and during the AN, respectively. The rate at SR was obviously far higher than the rates at the remaining daytimes (35 ± 3 workers/colony/3 min), with a value 2.4, 5.8, 6.0 and 2.7 times greater than the values of FN, N, AN, and SS, respectively. The colonies obtained 47.1%, 19.4%, 8.1%, 7.9% and 17.6% of their daily pollen income during SR, FN, N, AN, and SS, respectively (Figure 5).

The hourly rates of pollen gathering in the native colonies culminated with three peaks at the following daytimes: SR, FN and SS, and N-AN. The colonies finished two quintuples of their daily pollen-gathering efforts during SR (45 ± 5 workers/colony/3 min). They collected 39.9%, 20.4%, 9.2%, 9.6% and 20.9% of their daily pollen crop at SR, FN, N, AN, and SS, respectively (Figure 5).

The maximum and minimum pollen-gathering rates (25 ± 3 and 1 ± 0 workers/colony/3 min, respectively) of the Carniolan colonies occurred at SR and AN, respectively. Pollen was collected 4.3, 15.6, 26.0 and 9.4 times more actively around SR than during FN, N, AN and SS, respectively. The colonies completed more than two-thirds of their daily pollen-gathering work during the SR period. In general, they fulfilled 69.3%, 16.3%, 4.4%, 2.7% and 7.3% of their daily, pollen-gathering, incoming returns during the SR, FN, N, AN, and SS periods, respectively (Figure 5).

The pollen-gathering rates for all tested colonies during months of the Acacia flow season were significantly (*p* ˂ 0.01) different. Each hour, during May, June, July, and August, each colony made 264, 300, 342 and 230 pollen-gathering flights, respectively (Figure 6).

The pollen-gathering rates of the native colonies during Acacia season months varied significantly (*p* ≤ 0.05). The maximum and minimum monthly rates (25 ± 3 and 19 ± 3 workers/colony/3 min, respectively) occurred during July and August, respectively. Each native colony received 433, 436, 504 and 372 pollen-gathering incoming flights each hour during May, June, July, and August, respectively (Figure 6).

The monthly pollen-gathering rates of the Carniolan colonies peaked significantly (*p* ≤ 0.05) three times. The highest peak occurred during June-July, while the other two peaks occurred during May and August. The pollen-gathering activity was 1.9, 1.1 and 2.1 times greater during July than during May, June, and August. The Carniolan colonies made 96, 164, 182 and 87 pollen-gathering flights each hour during May, June, July and August, respectively (Figure 6). The native honeybee colonies preferred pollen gathering more than the Carniolan colonies, with ratios of 0.20 and 0.15, respectively.

### 3.4. Correlation between Foraging, Pollen Gathering and Weather Factors

Temperature was significantly (*p* ˂ 0.01) correlated with RH and WS (−0.774 and 0.437, respectively). RH and WS were also significantly (*p* ˂ 0.01) correlated (−0.517) with each other. The pollen-gathering rate was significantly (*p* ˂ 0.01) correlated with the foraging rate in both native and imported colonies (0.635 and 0.576, respectively). Consequently, the correlation coefficient factor was significantly (*p* ˂ 0.01) positive between the two activities (0.605) when the general mean of the two subspecies was considered. The foraging and pollen-gathering rates were significantly (*p* ˂ 0.01) correlated with the environmental weather factors (Table 1).

## 4. Discussion

This study resulted in five key findings: (1) the bee colonies collected food relatively well during Acacia flow, despite the extremely hot-dry and relatively windy environment; (2) the indigenous colonies were significantly more active than the imported colonies in food collection during Acacia flow under harsh desert weather; (3) a higher portion of all field trips was allocated to pollen gathering by the native bees than by the imported bees; (4) the nectar-rich Acacia trees reduced the adverse impacts of the hot-dry-windy weather; and (5) the foraging and pollen-gathering activities of the honeybees were affected by both weather conditions and the nectar-secretion rates of the present honeybee flora. Consequently, honeybee activities varied hourly, monthly, and seasonally according to variations in weather and Acacia nectar-secretion rates.

The honeybee colonies foraged and gathered pollen relatively well during Acacia flow. The present values are similar to the values that have been reported for non-hot-dry weather [24]. This relatively robust food-gathering rate suggests that the colonies produced a reasonable yield of Acacia honey. However, this conclusion needs to be experimentally confirmed. Food was collected significantly (*p* ≤ 0.05) more actively by native bees than by Carniolan bees during Acacia flow. The foraging rate of native honeybees was 2.2 times greater than that of imported honeybees. Furthermore, 3.1 times more pollen was gathered by the native colonies than by the imported colonies. These results are in agreement with previous studies [23,24]. The native bees proved well adapted to the hot-dry weather dominating their home land, the Arabian Peninsula. Alqarni [14] and Abou-Shaara, et al. [36] interpreted this adaptation as being due to morphological and physiological traits.

Nevertheless, the superiority of the native subspecies during Acacia flow could be partly due to their possible adaptation to the Acacia flower arrangement. Other potential features, such as different work-force build-up and swarming strategies, mediated largely by familiarity with resource availability, cannot be ignored for such differential performance. It is further assumed that the feeding behavior and niche compatibility of native honeybees have evolved to the morphology and phenology of Acacia flower heads. 

The present study indicated that the ratio of pollen gathering to foraging was 0.20 and 0.15 for the native and imported colonies, respectively. This means that a native colony allocates 20% of its field trips to pollen harvesting, while an imported colony allocates only 15% of its field trips to this activity. This preference could possibly be due to a high-pollen-hoarding genotype [37,38,39] in the native subspecies. This behavior has been described in other honeybee genotypes, such as some commercial strains of *A. mellifera* [40], Africanized honeybees [41,42] and *Apis cerana* [43]. However, high-pollen-hoarding has never been genetically investigated. Beekeepers have long noticed this behavior and have frequently described it in native bee colonies. Overall, further genetic, behavioral, and ecological studies on native *A.m. jemenitica* need to be conducted.

In the middle of the desert, under extremely hot-dry conditions of Temp ranging from 23 to 52 °C and RH values of 5–21%, bees are unable to forage unless a nectar-rich plant is accessible. Hence, the foraging activity of the tested bee colonies was greatly enhanced by the nectar-rich Acacia trees. The results show that the ascending trend in the monthly foraging rate during Acacia flow was temporally associated with the ascending rate of nectar secretion by the Acacia flower, as reported by Alqarni, Hassan and Owayss [20] in the same location. Meanwhile, the monthly means for the weather factors did not show clear differences during the same Acacia flow period. This implies that the monthly increase in the foraging rate from May through August could be due to the monthly increase in nectar secretion. In a nearby area with no *A. gerrardii* trees, Abou-Shaara, Al-Ghamdi and Mohamed [23] reported foraging activities of 73 and 43 workers/colony/5 min, which were less than the 109 and 49 workers/colony/3 min found in the present study for native and imported colonies, respectively, confirming the effect of the nectar-rich Acacia flow in this study. Additionally, Silva, et al. [44] suggested that the field trips of foraging honeybees may peak at the time of the day when abundant food resources are accessible.

Foraging and pollen-gathering activities were negatively affected by Temp and WS and positively affected by RH. The effects of weather on these two activities have been explored in previous studies [9,10,12,14,23]. In contrast to our results, Alamu, Omoayena and Amao [3] reported that the honeybee outgoing rate was positively and negatively affected by Temp and RH, respectively. Abou-Shaara, Al-Ghamdi and Mohamed [23] reported a weak correlation between foraging activity and both Temp and RH. Moreover, Omoloye and Akinsol [13] and Alamu, Omoayena and Amao [3] reported that honeybee workers grazed well during windy times, which was attributed to the good fragrance distribution under such conditions. The correlation of bee foraging and pollen-gathering activities with Temp, RH or WS should be positive if these factors are mostly below the optimum range and vice versa. Therefore, correlations between foraging and pollen-gathering activities and weather factors are logically expected to vary geographically, seasonally, and even hourly. On the other hand, the harsh summer weather of Central Arabia affects nectar secretion [26]. This could represent an indirect effect of harsh weather on honeybee foraging rates.

Monthly variations in foraging and pollen-gathering activities were more distinct than monthly weather variations. At least part of the monthly variations in foraging and pollen-gathering activities could be attributed to the monthly variations in the reward quantity of Acacia flowers, as reported by Alqarni, Hassan and Owayss [20]. As weather factors affect nectar-secretion rates [18,45], they could also affect the activities of bees by affecting their reward.

More studies are needed to explore the honeybee–Acacia interaction and to evaluate how much honey a bee colony could produce during Acacia flow. More specifically, the behavior of bee workers on the flower heads of Acacia needs to be described, as well as the strength and production of the colony during Acacia flow.

In conclusion, bee colonies successfully collected food in a subtropical region during *A. gerrardii* rich nectar flow, despite the coincident hot-dry-windy weather. The native honeybee subspecies more actively foraged and gathered pollen than the Carniolan colonies, an indication that the former honeybee is better adapted than the latter honeybee to benefit from the Acacia reward and to pollinate Acacia flower heads. Nectar-rich bee flora can enhance the foraging rates of honeybee colonies for the benefit of their nectar, overcoming the hot-dry-windy environments in subtropical ecosystems.

## 5. Conclusions

The native honey bees (*Apis mellifera jemenitica*) were found more active with better foraging rate, food and pollen gathering than Carolina bees (*Apis mellifera carnica*) in the presence of nectar-rich Acacia trees in the desert of central Arabia under drastic weather conditions. The nectar-rich flora positively reduced the negative effects of hot, dry and windy weather in the subtropical conditions and resulted in the better performance of honey bees. 

## Figures and Tables

**Table 1:** Pearson correlation coefficient factors between foraging and pollen-gathering rates of indigenous (*Apis mellifera jemenitica*) and exotic (*A.m. carnica*) honeybee colonies and weather factors during the *Acacia gerrardii* flow (May–August 2017–2018) in Rawdhat-Khoraim, Riyadh, Saudi Arabia.

Subspecies	Activity	Temp.	RH	Wind Speed
**Indigenous**	**Foraging**	−0.357 ^**^	0.297 ^**^	−0.335 ^**^
	**Pollen gathering**	−0.301 ^**^	0.289 ^**^	−0.207 ^**^
**Exotic**	**Foraging**	−0.426 ^**^	0.293 ^**^	−0.319 ^**^
	**Pollen gathering**	−0.449 ^**^	0.347 ^**^	−0.240 ^**^
**Mean**	**Foraging**	−0.424 ^**^	0.325 ^**^	−0.362 ^**^
	**Pollen gathering**	−0.390 ^**^	0.339 ^**^	−0.240 ^**^

** refers to a highly significant (*p* ˂ 0.01) correlation.
